# Statistical analysis and estimation of the regional trend of aerosol size over the Arabian Gulf Region during 2002–2016

**DOI:** 10.1038/s41598-018-27727-0

**Published:** 2018-06-22

**Authors:** Alina Barbulescu, Yousef Nazzal, Fares Howari

**Affiliations:** 1 Ovidius University of Constanta, Constanta, Romania; 2 College of Natural and Health Sciences, Zayed University, Abu Dhabi, United Arab Emirates

## Abstract

In this article, we present the results of the regional estimation of the evolution of monthly mean aerosol size over the Arabian Gulf Region, based on the data collected during the period July 2002 – September 2016. The dataset used is complete, without missing values. Two methods are introduced for this purpose. The first one is based on the partition of the regional series in sub-series and the selection of the most representative one for fitting the regional trend. The second one is a version of the first method, combined with the k-means clustering algorithm. Comparison of their performances is also provided. The study proves that both methods give a very good estimation of the evolution of the aerosol size in the Arabian Gulf Region in the study period.

## Introduction

Aerosols are tiny particles suspended in the atmosphere which result from natural or anthropic sources. The natural aerosols are classified in: product of sea spray evaporation, mineral aerosol, volcanic aerosol, particles of biogenic origin, smokes from burning on land, sulphates^1^.

Ginoux *et al*.^2^ did an extensive study for attribution of anthropogenic and natural dust sources. Haywood *et al*.^3^ indicate that the aerosols cause a strong radiative forcing of climate because of their efficient scattering of solar radiation. Since they are acting as cloud and ice concentration nuclei, the aerosols have a significant impact on the climate variation^4^. Their diameters vary from less than one nanometer to 100 µm, those of the natural aerosols being generally higher than those of the human-made ones^5^. Particles larger than about 1 μm are also called coarse particles.

One of the most abundant aerosols in the atmosphere is the dust. Desert dust particles, also called mineral aerosol, are soil particles suspended in the atmosphere in the area with easily erodible dry soils, strong winds and little vegetation^5^. They are composed of oxides (silica, iron oxides), quartz, feldspar, gypsum and hematite etc^1^. Their inhalation is dangerous for the human health because they can get deposited in the gas-exchange region of the lungs^6,7^. Therefore, their production and transport must be investigated.

Scientists found that dust particles enter the lower atmosphere primarily through a mechanism called saltation bombardment, which is strongly dependent on the meteorological conditions near the surface, as well as on the soil texture and particle size^8,9^. According to Astitha *et al*.^10^, massive amounts of dust are lofted in deep atmospheric boundary layers over the hot deserts. Spyrou *et al*.^11^ emphasized that extensive plumes can travel across multiple countries at high altitudes up to the middle troposphere, while other scientists emphasized the transport of the aerosol from Africa and Asia^12–14^. Levin *et al*.^15^ analyzed the interaction mechanism between the mineral dust, sea-salt particles, and clouds, while Smoydzin *et al*.^4^ analyzed the role of the meteorological conditions on the pollution over the Arabian Gulf. They stated that the dust is emitted as hydrophobic particles, relatively ineffective as cloud condensation nuclei, but during their transport in the atmosphere, and the interaction with gaseous and particulate air pollutants, their hygroscopicity increases, enhancing the efficiency of the dust removal through precipitation^16^. Alfaro and Gomes^17^ proposed a model for mineral aerosol production by wind erosion by combining preexisting models of saltation and sandblasting processes that lead to mineral aerosol release in arid areas, while the impact of dust particles on the condensation nuclei, important in the precipitation formation and its spatial distribution has been analysed by Karydis *et al*.^18^.

Namikas^19^ investigated the causes of wind erosion in sand dunes. His research revealed that wind speed varies regionally, frequently occurring during certain periods and its interaction with the land-use disturbances may produce big quantities of atmospheric dust. It was shown that much of the desert dust mass transported in the atmosphere occurs during a few events. Part of them, occurring in South and Central Asia have been extensively studied by Chen *et al*.^12^ and Huang *et al*.^20^.

Recent research indicates a significant variability of the airborne desert dust concentrations that during the past decades in the Middle East, Africa, Central Asia and South America^12,21–23^, and the augmentation of aerosol optical depth worldwide, especially in spring and summer, suggesting a relationship with the dust abundance^24^. Analyses of airborne desert dust and atmospheric concentrations of mineral dust particles extending from the Sahara, across the Arabian Peninsula and the Middle East, to South and Central Asia have been provided, as well^2,13,14,20^.

Most article focused on classifying the aerosol types using the satellite data. The classification are based on the aerosol optical depth^25,26^, Angstrom exponent and index of refraction^27–29^, or fine mode fraction and the aerosol index^30^.

The reviews on the impact of the dust size on climate and biogeochemistry, are focusing on the characterization of the size distributions of the aerosol particles. It was shown that the particles with sizes between 0.2–2 *μ*m produce the largest shortwave radiative effect per unit mass^20,31^. As condensation nuclei, the number of particle above a given size is important^32^. Point of view of geochemistry, the amount of dust deposition is essential.

Given the importance of the study of aerosols dimensions, we notice only few articles treating this topic^20,33^.

Therefore, we aim at analyzing the series of aerosols radius over the Arabian Gulf Region and to model the trend of the regional series. Two approaches are proposed, based on the partition of the regional series into subseries and the selection of the most representative one, used for building the regional trend.

In the following, we shall understand by *particular series*, a series recorded at a certain point and by the *regional series*, the multidimensional series containing all the particular series.

## Results and Discussions

**Figure 1:**
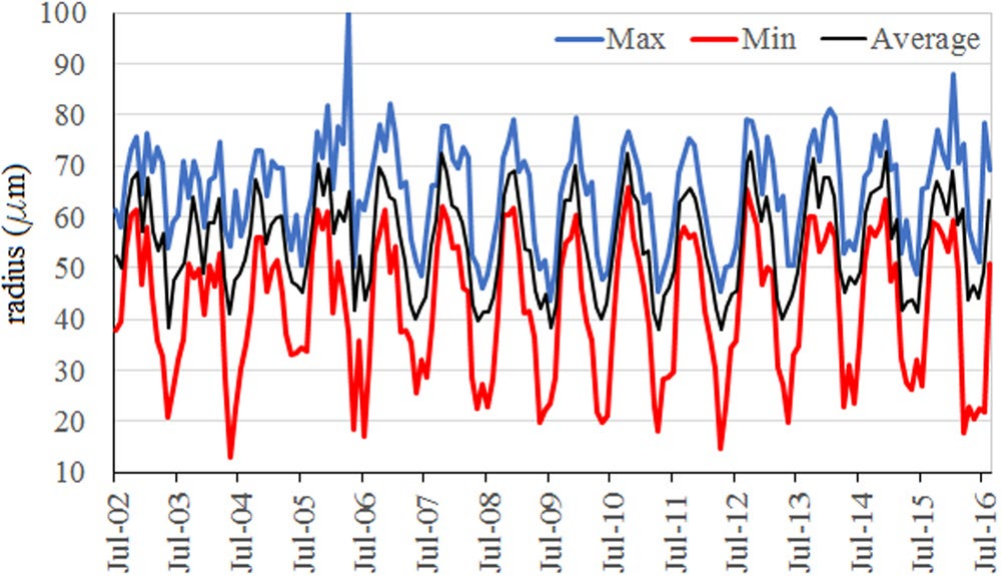
Maxima, minima and the average series during the study period.

**Figure 2:**
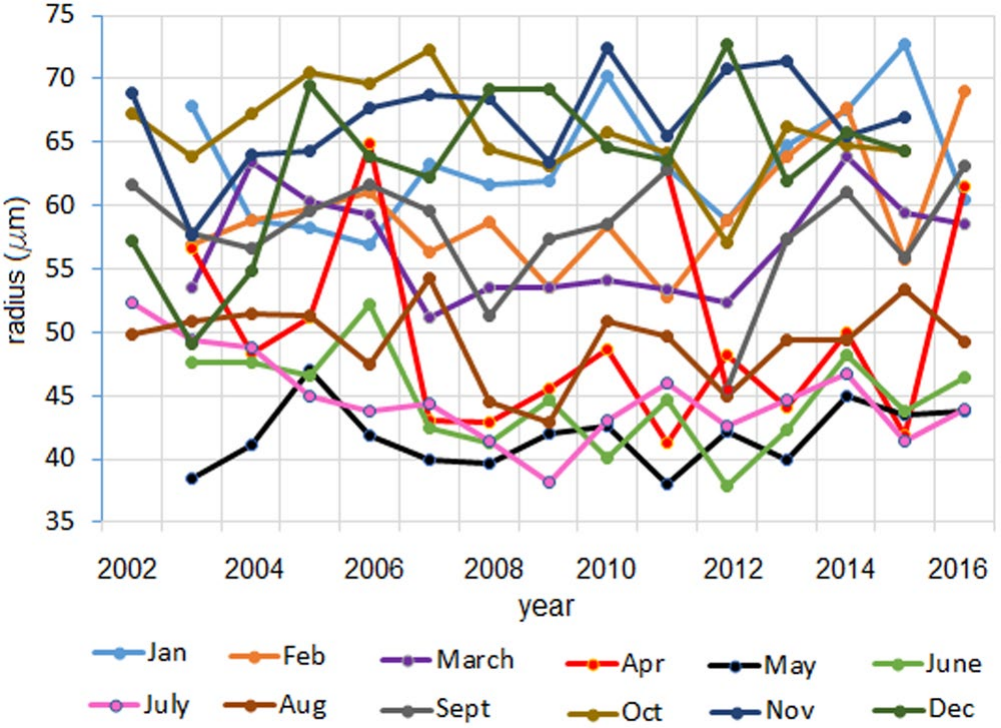
Regional monthly average series during the study period.

Figure 1 represents the maxima, minima and average regional series during the study period and Fig. 2 presents the monthly average of the series in the study period.

We remark a seasonal variation of the series. At the beginning of the period the highest means have been recorded in October, November and December, but after 2010, we notice the highest monthly averages in November, December and January. The months when the smallest aerosols radii have been registered are May, June and July.

The analysis of the aerosols’ radii at all the observation sites provide the following limits: the minimum is between 12.99 and 39.76 µm, the maximum is between 71.65 and 100 µm, the average is in the interval 51.21–56.79 µm, with the standard deviation in the interval 8.67–15.21. The series’ skewness is between −0.41 and 0.48, 39% being in the interval [−0.05, 0.05], 31% greater than 0.05 and 33% less than −0.05, indicating that the majority of the series distributions are left or right skewed. The excess kurtosis is between −1.31 and 1.05: only 5 values are greater than zero, and approximately 90% around −1. Therefore the majority of the distributions of the dust aerosol size are platykurtic, only five of them being leptokurtic.

The normality hypothesis for the individual series has been rejected for all the series by the Shapiro-Wilk and Anderson-Darling tests. Jarque-Bera’s test rejected the normality hypothesis for 95% of series. This result is in concordance with the finding related to the skewness and excess kurtosis.

After performing the Henze – Zirkler test, the null hypothesis was rejected as well. Therefore, the regional series does not follow a multivariate Gaussian distribution.

After applying the dcor t-test, the independence hypothesis has been rejected since the dcor coefficients are higher than 0.586. Since the p-value associated with the Kruskall-Wallis test is less than 0.0001, we can’t accept the hypothesis that all the samples come from the same population. Therefore, the series are not independent, but they don’t have the same distribution.

For emphasising the differences between the pairs of series, after the rejection of the null hypotheses by the Kruskall-Wallis test, the post-hoc Dunn’s test was performed. Its results show that 82.2% of pairs of series don’t come from the same population.

**Figure 3:**
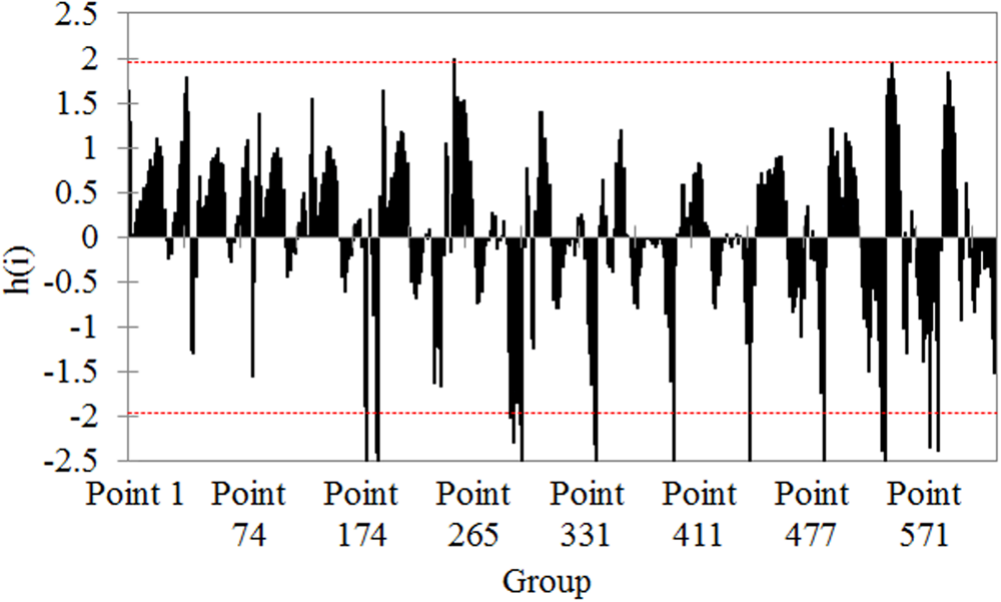
Results of Mandel’s test.

Mandel’s test has been performed for detecting if there are outlying means among the series averages. It was found that there are only 18 outlying means of the study series. Figure 3 illustrates the result of this test. The dotted lines represent the critical values of the test (1.956 and −1.956), and the vertical lines, the values of h-statistic for the individual series. Most of these values are in the range from −1.25 to 1.25.

The Brown-Forsythe test rejected the homoscedasticity hypothesis. Therefore, we can conclude that the variances of the individual series are not homogenous.

For modeling the regional distribution of the aerosols’ size, we used the following two algorithms.

### Method I

1. Given the data series recorded at *m* different observation points, on *n* consecutive periods, build the matrix of the regional series, $$Y={({y}_{ji})}_{\begin{array}{l}j=\overline{1,n}\\ i=\overline{1,m}\end{array}}$$, where *y*_*ji*_ is the series recorded at the moment *j* at the point *i*. So, a column of the matrix *Y* contains the data collected at a specific point, and a raw of the matrix contains all the values collected at a certain moment at all the sites.
  For each $j=\overline{1,\,n}$, perform the steps (2)–(6).
2. Compute the extreme values (maximum and minimum) on each row of the matrix (that is the regional extrema at each moment) and the amplitude, as the difference between the maximum and the minimum values.
  For example, denoting respectively by *y*_*j* max_ and *y*_*j* min_ the maximum and minimum values at the moment *j*, the amplitude at the moment *j* will be defined by:
  1 $${A}_{j}={y}_{j{\rm{\max }}}-{y}_{j{\rm{\min }}}$$
3. Divide the intervals [*y*_*j* min_, *y*_*j* max_] into a convenient number of sub-intervals, *m*_*j*_, of length *L*_*j*_ = *A*_*j*_/*m*_*j*_, such as each sub-interval contains enough values.
  Let us denote by *I*_*jl*_ the sub-interval *l* of the period *j*.
4. Attach to each sub-interval interval *I*_*jl*_ its frequency, *f*_*jl*_, defined as the number of values from *I*_*jl*_.
5. Choose that interval *I*_*jl*_ whose frequency is maximum. Denote it by *I*_*j* max_ and by *f*_*j* max_ the corresponding frequency. If the highest frequency appears more than once, *I*_*j* max_ will be that interval whose average is the closest to the average of the entire period *j*. For example, suppose that the maximum frequency is *f*_*j*3_ = *f*_*j*5_, the average of the values in *I*_*j*3_ is 24.5, the average of the values in *I*_*j*5_ is 29.5 and the average of the period *j* is 30. Then, we select *I*_*j* max_ = *I*_*j*5_ because 29.5 is closer to 30 than 24.5.
6. Choose the representative value for the period *j* to be equal to the average of the values from the interval *I*_*j* max_, and denote it by ${\overline{y}}_{j\max }$.
7. Build the trend series that fit the regional one using ${({\overline{y}}_{j\max })}_{j=\overline{1,n}}$.
8. Estimate the fitting quality, computing the mean absolute error and the mean standard error of each series *i* $(i=\overline{1,m})$^34^.

The mean absolute error and the mean standard error of the series *i* (denoted respectively by MAE_*i*_ and MSE_*i*_) are defined by:

2 $${{\rm{MAE}}}_{i}=\frac{1}{{n}_{i}}\sum _{q=1}^{{n}_{i}}|{x}_{iq}-{({x}_{iq})}_{e}|,$$

3 $${{\rm{MSE}}}_{i}=\sqrt{\frac{1}{{n}_{i}}\sum _{q=1}^{{n}_{i}}{[{x}_{iq}-{({x}_{iq})}_{e}]}^{2},}$$

where *n*_*i*_ is the number of values of the series *i*, *x*_*iq*_ is the *q*^th^ value of the series *i*, (*x*_*iq*_)_*e*_ is the value estimated by the model for the *q*^th^ value of the series *i*.

Lower MAE_*i*_ and MSE_*i*_ are, better the modeling quality is.

### Method II

This algorithm is based on the previous one, but the selection of the interval with the maximum frequency is replaced by the selection of the cluster with the highest number of elements, after pruning the k-means clustering algorithm, for classifying the series in clusters (homogeneous and disjoint groups within which the patterns are similar). The number of clusters, k, is a priori specified and the algorithm k-means stores k centroids used to define clusters.

The clustering idea is finding groups (clusters) that minimise an error criterion, as, for example, Sum of Squared Error (SSE), which measures the total squared Euclidian distance of instances to their representative values^35^.

Considering Z = (*z*_1_, *z*_2_, …, *z*_*m*_), *z*_*i*_ ∈ ***R***^*n*^, $i=\overline{1,m}$ being the given points, the k-means clustering algorithm has the following steps^32^:

- Choose the number of clusters, k.
- Initialize the cluster centroids *v*_1_, *v*_2_, …, *vk* ∈ **R**^*n*^ randomly.
- Compute the distance between each data point and the cluster centers.
- Assign the data point to the cluster whose distance from the cluster center is the minimum of all distances to the cluster centers.
- Compute the new cluster center by
  4 $${v}_{i}=\frac{1}{{c}_{i}}\sum _{j=1}^{{c}_{i}}{z}_{j},$$
  where *c*_*i*_ is the number of the data points in *i*^*th*^ cluster.
- Restart from (b) till no data point will be reassigned to the cluster. Then stop.

For more information about other clustering algorithms, the reader could refer to Rokach and Maimoon^36^, Everitt *et al*.^37^, Xu and Wunsch^38^.

In our case, *Z* = *X* and the element *z*_*i*_ is the column *i* of the matrix *X*.

The stages or *Methods II* are the following:

(I) Similar to step (1) of the Method I.
(II) Choose the number of clusters, *k*, and perform the *k*-means clustering.
(III) Determine the cluster with the highest number of elements and build the matrix *X*_*c*_ with the columns of *X* that contain the values recorded at the observation points included in this cluster.
(IV) Choose the representative value for the period *j* to be equal to the average of the values from the *j*^*th*^ row of *X*_*c*_. Denote it by ${\overline{y}}_{jC}$.
(V) Build the series that fit the regional one, using ${({\overline{y}}_{jC})}_{j=\overline{1,n}}$, and estimate the fitting quality by computing MAE_*i*_ and MSE_*i*_ of each series $i=\overline{1,m}$.

The comparison of the methods’ performances is done by computing the mean absolute error and mean standard error of each series and the overall the mean absolute error and mean standard error.

The overall mean absolute error is defined by:

5 $${\rm{MAE}}=\frac{1}{\sum _{i=1}^{m}{n}_{i}}\sum _{i=1}^{m}({n}_{i}\cdot {{\rm{MAE}}}_{i}),$$

and the overall mean standard error is given as:

6 $${\rm{MSE}}=\sqrt{\frac{1}{\sum _{i=1}^{m}{n}_{i}}\sum _{i=1}^{m}({n}_{i}\cdot {{\rm{MSE}}}_{i})},$$

where MAE is the overall mean absolute error, MSE is the overall mean standard error, MAE_*i*_ is the mean absolute error of the series *i*, MSE_*i*_ is the mean standard error of the series *i, n*_*i*_ is the number of values in the series *i*.

Lower MAE (or MSE) is, better the modeling result is.

The modeling techniques have been applied for monthly average series collected at the study sites for 171 month. The data series and the computation done by applying Methods I and II can be found in the Supplementary Dataset 1 and Supplementary Tables S1 and S2.

For Method I, *n* = 387, *m* = 171. We chose *m*_*j*_ = 6, for all $j=\overline{1,171}$ at stage (3), meaning that after detecting the range of the values in a given period, this interval was divided into 6 subintervals. Method II was applied using a number of clusters *k* = 6, for comparison reasons.

After performing the k - means algorithm we got the results presented in Tables 1–3.

**Table 1:** Distances between the class centroids.

Class	1	2	3	4	5	6
1	0	54.557	62.361	57.625	46.242	58.259
2	54.557	0	36.057	59.761	28.393	38.034
3	62.361	36.057	0	45.503	43.235	23.329
4	57.625	59.761	45.503	0	54.052	42.183
5	46.242	28.393	43.235	54.052	0	40.089
6	58.259	38.034	23.329	42.183	40.089	0

**Table 2:** Distances between the central objects.

Central object	1 (Point 57)	2 (Point 88)	3 (Point 160)	4 (Point 242)	5 (Point 283)	6 (Point 296)
1 (Point 57)	0	62.988	74.500	72.723	55.341	67.226
2 (Point 88)	62.988	0	49.429	69.936	37.787	49.918
3 (Point 160)	74.500	49.429	0	60.791	55.932	34.831
4 (Point 242)	72.723	69.936	60.791	0	63.364	53.695
5 (Point 283)	55.341	37.787	55.932	63.364	0	49.296
6 (Point 296)	67.226	49.918	34.831	53.695	49.296	0

**Table 3:** Results of the k-means clustering algorithm by class.

Class	1	2	3	4	5	6
Objects’ number	19	105	111	50	50	52
Within-class variance	2935.890	821.017	870.805	1950.387	1547.098	781.011
Minimum distance to centroid	36.693	17.443	19.743	24.940	19.993	17.046
Average distance to centroid	50.604	27.306	28.408	41.550	36.373	26.409
Maximum distance to centroid	95.749	61.838	59.983	77.899	82.159	51.011

We remark that the within-class variances, minimum, maximum and average distances to centroids are close to each other for the classes 2 and 3. The classes have different numbers of elements, the highest one being in the third one that will be selected for modeling with Method II.

**Figure 4:**
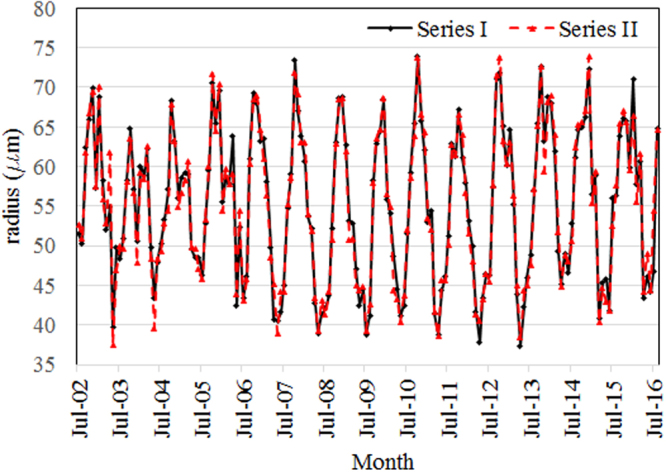
Models obtained by Method I (Series I) and Method II (Series II).

The regional series obtained by applying the methods previously described are presented in Fig. 4, where Series I is obtained by Method I and Series II is obtained by Method II.

**Figure 5:**
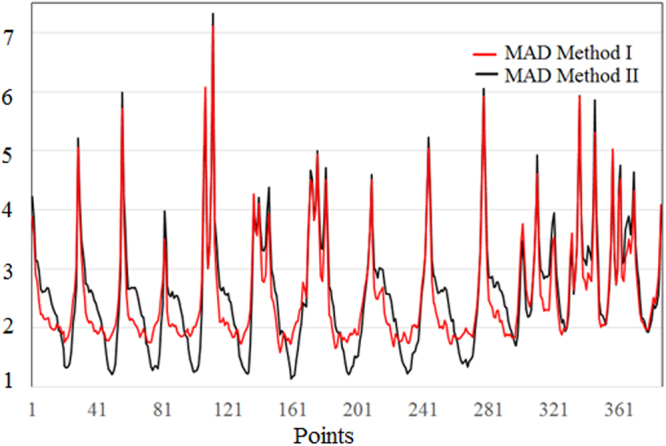
Mean absolute error (MAD) in the models.

**Figure 6:**
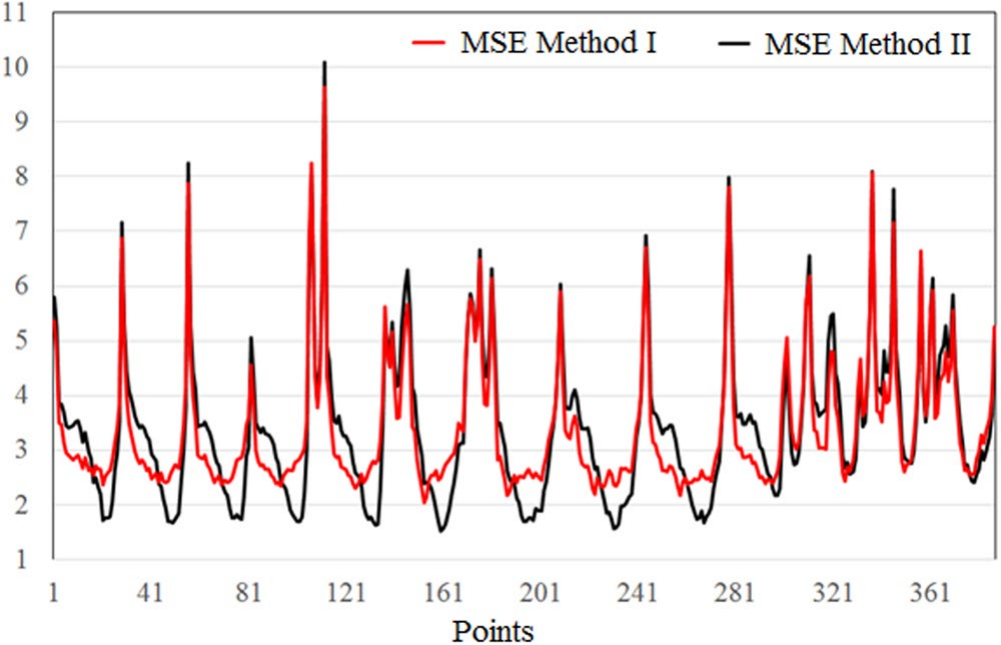
Mean standard error in the models.

The corresponding errors (MAD and MSE) are presented in Figs 5 and 6. They were obtained based on the data provided in the Supplementary Tables S1 and S2. The values from the Supplementary Tables S1 and S2 can be synthetized in Table 4, where the minimum and maximum modeling error are presented.

**Table 4:** Comparison of modeling errors.

Method	*MAD*_*i*_		MAD	*MSE*_*i*_		MSE
	min	max		min	max	
I	1.584	7.111	2.536	2.040	9.629	3.547
II	1.137	7.730	2.607	1.514	10.083	3.659

Mean absolute deviations and mean standard error of the individual series (*MAD*_*i*_, *MSE*_*i*_) vary in larger limits when using Method II, than when using Method I. Comparing the MADs (respectively MSEs) from Supplementary Tables S1, one can see that the first method better performed for 223 series (respectively 218 series). This means that MADs (respectively MSEs) were smaller in 57.62% (respectively 56.33%) cases when Method I was used. Overall MADs for the models were respectively 2.536 and 2.607. Overall MSEs for the models were respectively 3.547 and 3.639. Therefore, the overall mean absolute deviations and mean standard errors are comparable for both methods.

The study series were not highly inhomogeneous. Only some outlying means were detected and the standard deviations were between 8.69 and 15.25. This could be a reason for which the goodness of fit of the methods are similar.

The Method I gave better results due to the procedure of selection of the values of the individual series that participate in fitting the regional one. While in the second method, all the values that participate to this process are taken from the same series, belonging to the cluster with the highest number of member, in Method I, the values taken into account at each moment can belong to different series. For example, the representative values for the time *t* = 2 could be in the subinterval 3 and those for the time *t* = 4 could be in the subinterval 6.

## Conclusions

In this article, we presented two methods for estimating the evolution of the regional time series of the aerosols dimensions. Both methods are easy to use, the advantage of the second one being that the k-mean algorithm is implemented in many software. In both methods, the number of subintervals, respectively clusters must be specified from the beginning for the selection of the series that will finally participate in fitting the regional series. The fitting results are not significantly different (in terms of overall MADs and MSEs), but the first method gave a better estimation of the individual series values (223 and 218 series, respectively). For highly inhomogeneous series, we recommend the use of the first method, knowing that the k-mean algorithm is sensitive to the outliers’ existence. Therefore statistical tests for the mean homogeneity and homoskedasticity, as well as that for the outliers’ existence must be performed before deciding the modeling method.

Both methods could be successfully used to estimate the regional evolution of the pollutants’ series when some particular series in the study area present missing values, but the neighboring series are complete. In the future works we shall study the sensitivity of both methods to the selection of the clusters’ number. We shall compare the methods’ goodness of fit for regional series of data (as precipitation, temperature, anthropic aerosol) before and after the seasonality removal, as well. We also aim at implementing both methods in a friendly - user software.

## Methodology

### Data series

**Figure 7:**
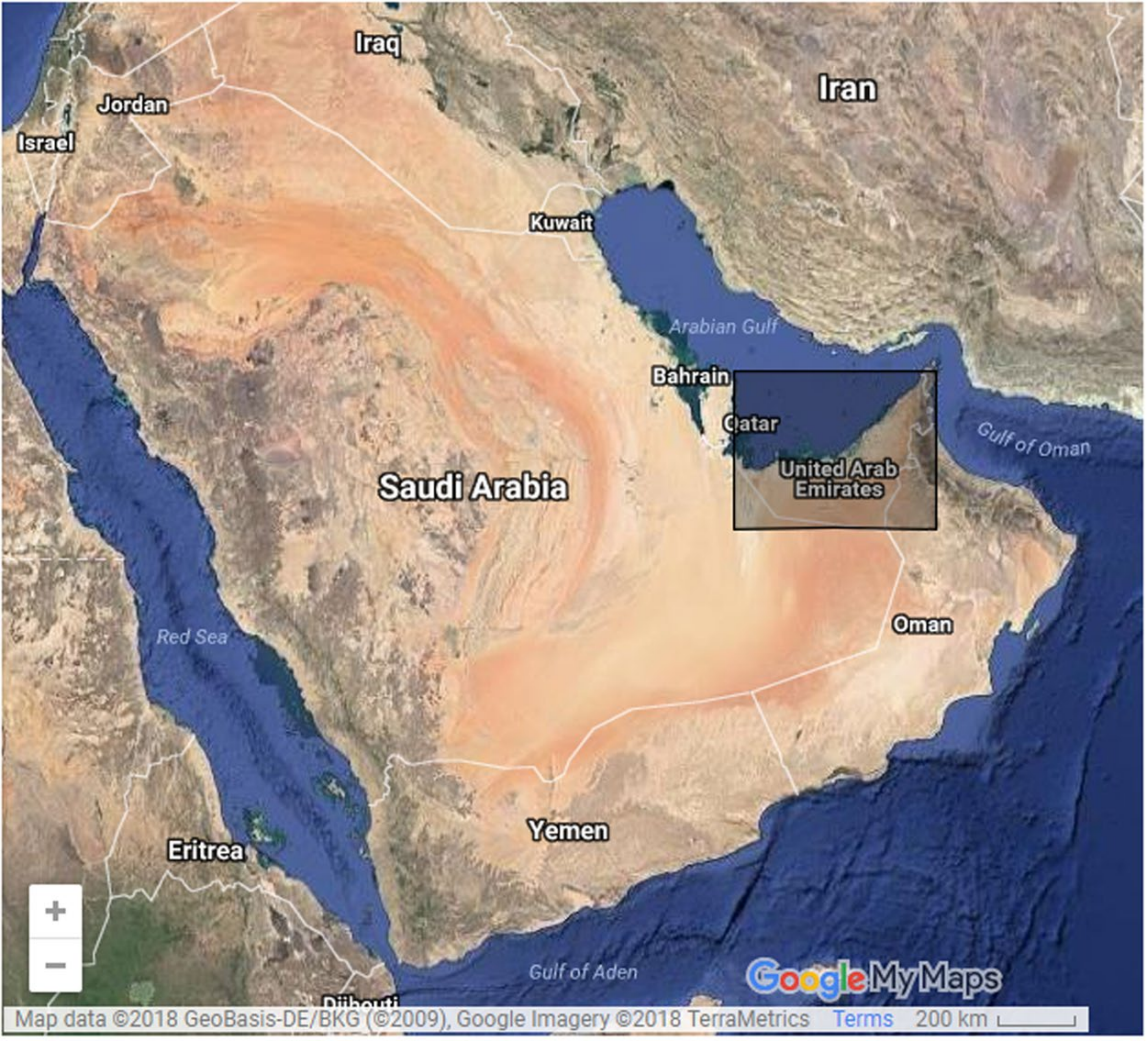
Observation area - inside the rectangle (https://www.google.com/mymaps/, 2018).

Data used are monthly series containing the aerosol particle radius record from July 2002 till September 2016, over the Gulf Region (Fig. 7), retrieved by MODIS^39^. There were 1850 observation points. For modeling the regional evolution of the dimension of aerosol particles we selected 387 complete series (without missing data), each of them containing 171 values. The coordinates of these points can be found in Supplementary Dataset.

To determine the type of aerosols, we compared the aerosols dimensions and the optical depth (AOT) downloaded from MODIS site^39^ for our series, with the results from the literature^25–29^. It resulted that the type of aerosol is mostly dust because AOT was larger than 0.3 (majority around 0.43). The aerosols’ dimensions are in the range of the coarse aerosols (as per the data from the Supplementary Dataset). Due to lack of space and since our main purpose is the modeling, we shall not insist on other characteristics of the study aerosols. We intend to dedicate another article to an extensive study of their properties.

### Statistical analysis

Before modeling, statistical analyses of the series have been performed, at the significance level *α* = 0.05. For all the tests, when the p-value associated is lower than the significance level, one should reject the null hypothesis H_0_, and accept the alternative hypothesis H_a_.

In what follows we shall call *individual series* the series recorded a certain observation point and the *regional series* that one recorded at all the sites. Therefore, an individual series will be represented by a column vector and the regional series as a matrix formed by all the columns containing the individual series.

The statistical tests have been conducted using the R software.

For testing the normality hypothesis against the non-normality for the individual series, the Anderson-Darling^40^, Jarque-Bera^41^ and Shapiro-Wilk^42^ tests were performed. They are implemented in ‘fBasics’ package in R^43^.

The test statistic for the Anderson-Darling test is defined as:

7 $${A}^{2}=-\,n-\frac{1}{n}\sum _{i=1}^{n}(2i-1)[\mathrm{ln}\,{p}_{(i)}+\,\mathrm{ln}(1-{p}_{(n-i+10)})],$$

where

8 $${p}_{(i)}={\rm{\Phi }}([{x}_{(i)}-\overline{x}]/s),$$

Φ is the cumulative distribution function of the standard normal distribution, $\bar{x}\,\,$is the mean and *s* is the standard deviation of elements in the sample,${\{{x}_{i}\}}_{i=\overline{1,n}}$.

If the p-value associated to the test is less than the significance level, the normality hypothesis can be rejected.

The Jarque-Bera test statistic is defined as:

9 $$JB=n(\frac{{k}_{3}^{2}}{6}+\frac{{k}_{4}^{2}}{24}),$$

where *n* is the sample volum, *k*_3_ is the sample skewness and *k*_4_ is the sample excess kurtosis, defined as:

10 $${k}_{3}=\frac{1}{n{s}^{3}}\sum _{i=1}^{n}{({x}_{i}-\bar{x})}^{3},$$

11 $${k}_{4}=\frac{1}{n{s}^{4}}\sum _{i=1}^{n}{({x}_{i}-\bar{x})}^{4}-3.$$

For large samples, the JB statistics is compared to a chi-squared distribution with 2 degrees of freedom (*χ*^2^(2)). The normality hypothesis is rejected if the test statistic is greater than *χ*^2^(2).

The statistic of the Shapiro-Wilk test is defined as:

12 $$W=\frac{{(\sum _{i=1}^{n}{w}_{i}{x^{\prime} }_{i})}^{2}}{\sum _{i=1}^{n}{({x^{\prime} }_{i}-\bar{x})}^{2}}$$

where *n* is the sample volume, *x*_1_, *x*_2_, …, *x*_*n*_ are the original data, ${x^{\prime} }_{1},{x^{\prime} }_{2},\mathrm{...},{x^{\prime} }_{n}$ are the ordered data, $\overline{x}$ is the sample mean of the data, and the constants *w*_*i*_ are given by:

13 $$({w}_{1},{w}_{2},\ldots ,{w}_{n})=\frac{{M}^{T}{V}^{-1}}{{({M}^{T}{V}^{-1}{V}^{-1}{M}^{T})}^{1/2}},$$

where *M* = ${({m}_{1},{m}_{2},\ldots ,{m}_{n})}^{T}$ (the transposed vector (*m*_1_, *m*_2_, …, *m*_*n*_)) formed by the expected values of the order statistics of independent and identically distributed random variables sampled from the standard normal distribution and *V* is the covariance matrix of those order statistics.

Small values of *W* indicate non – normality.

All software provide the p-values corresponding to this test statistics. If the p-value is less than the significance level, the normality hypothesis can be rejected.

For testing the multivariate normality (the normality of the regional series) against the non-normality hypothesis, we used the multivariate Henze - Zirkler test^44^, implemented in the R package ‘MVN’^45^. The test is presented in the following.

Let *X*_1_, *X*_2_, …, *X*_*n*_ ∈ **R**^*d*^ be a random sample, where *d* is the dimension of *X*_*i*_ and *n* is the number of observations.

The Henze - Zirkler test is based on a nonnegative functional *D* that measures the distance between two distribution functions and has the property that *D*(*N*_*d*_(0, *I*_*d*_), *Q*) = 0 if and only if *Q* = *N*_*d*_(0, *I*_*d*_), where *N*_*d*_(*μ*, ∑_*d*_) is a *d*-dimensional normal distribution.

The test statistic *T*_*β*_(*d*) is defined by:

14 $$\begin{array}{rcl}{T}_{\beta }(d) & = & \frac{1}{{n}^{2}}\sum _{j=1}^{n}\sum _{k=1}^{n}{\exp }(-\frac{{\beta }^{2}}{2}{|{{Y}}_{j}-{Y}_{K}|}^{2})-2{(1+{\beta }^{2})}^{-d/2}\frac{1}{{n}}\\  &  & \times \sum _{j=1}^{n}{\exp }(-\frac{{\beta }^{2}}{2(1+{\beta }^{2})}{|{Y}_{j}|}^{2}+{(1+{\beta }^{2})}^{-d/2}),\end{array}$$

where

15 $$\beta =\frac{1}{\sqrt{2}}{(\frac{2d+1}{4})}^{1/(d+4)}{n}^{1/(d+4)},$$

16 $$\,{|{Y}_{j}-{Y}_{k}|}^{2}=({X}_{j}-{X}_{k})\text{'}{{\boldsymbol{S}}}^{-1}({X}_{j}-{X}_{k}),$$

17 $$\,{|{Y}_{j}|}^{2}=({X}_{j}-\bar{X})^{\prime} {{\boldsymbol{S}}}^{-1}({X}_{j}-\bar{X})$$

and **S** is the sample covariance matrix of the matrix **X** formed by ${X}_{1},\,{X}_{2},\ldots \,,\,{X}_{n}$ as columns.

In the null hypothesis, the test statistic is approximately lognormal distributed. The null hypothesis is rejected if the p-value associated to the test statistics is less than the significance level.

Since the regional series is not multivariate Gaussian, for testing the independence hypothesis of the regional series, the nonparametric dcor t-test^46^ of independence has been performed.

The test statistics is:

18 $${T}_{n}=\sqrt{\nu -1}\cdot \frac{{R}_{n}^{\ast }}{\sqrt{1-{({R}_{n}^{\ast })}^{2}}},$$

where *n* is the sample size, ${R}_{n}^{\ast }$ is the distance correlation statistics and $\nu =n(n-3)/2$.

The test rejects the null hypothesis at level α if ${T}_{n} > {c}_{\alpha }$, where ${c}_{\alpha }\,\,$is the (1−α) quantile of a Student *t* distribution with ν − 1 degrees of freedom. For details, the reader may refer to the article of Székely and Rizzo^46^.

For testing the hypothesis *H*_0_*: The individual series come from the same population* against the alternative *H*_*a*_*: The individual series do not come from the same population*, the Kruskall-Wallis test^47^ was performed.

The first stage all data is ranked, ignoring the group membership. The rank of the tied values is the average of the ranks they would have received had they not been tied.

The test statistic is defined as:

19 $$H=(N-1)\frac{\sum _{i=1}^{k}{n}_{i}{(\overline{{r}_{i.}}-\bar{r})}^{2}}{\sum _{i=1}^{k}\sum _{j=1}^{{n}_{i}}{({r}_{ij}-\bar{r})}^{2}},$$

where$\,N$ is the total number of values, *k* is the number of groups, ${n}_{i}$ is the number of observations in the group $i$, ${r}_{ij}$ is the rank of observation *j* from group $i$, among all observations, $\overline{{r}_{i}}$ is the mean of the ranks of the observations in the group $i$, $\bar{r}=(N+1)/2.$

If the null hypothesis is true, *H* has approximately a chi-square distribution with *k* − 1 degrees of freedom, ${\chi }_{k-1}^{2}.$ Therefore, the null hypothesis is rejected if ${\rm{H}} > {\chi }_{\alpha ;\,\,k-1}^{2},\,\,$found in the tables of the chi-square distribution at the significance level $\alpha \,$(generally set to be 0.05).

If the null hypothesis is rejected, the multiple pairwise comparisons are done using the Dunn’s test^48^.

Let *W*_*i*_ the *i*^th^ group’s summed ranks and *n*_*i*_ its sample size and $\overline{{W}_{i}}={W}_{i}/{n}_{i}$. Assign any tied values the average of the ranks they would have received had they not been tied.

For testing the hypothesis that the group *A* and *B* come from the same population against the hypothesis that they don’t come from the same population, we compute

20 $${z}_{i}=\frac{\overline{{W}_{A}}-\overline{{W}_{B}}}{{\sigma }_{i}}$$

where:

21 $${\sigma }_{i}=\sqrt{(\frac{N(N+1)}{12}-\frac{\sum _{s=1}^{r}{\tau }_{s}^{3}-{\tau }_{s}}{12(N-1)})(\frac{1}{{n}_{A}}+\frac{1}{{n}_{B}})},$$

*N* is the total number of elements in all the *k* groups,

*r* is the number of tied ranks across all the groups,

${\tau }_{s}\,\,$is the number of elements across all the groups, with the s^th^ tied rank.

If there are no ties, the second term in the first bracket is zero.

Denote by *k* the number of groups and ${z}_{1-\alpha /2k}$, the (1 −*α/2k*) point of the standard normal distribution. If $|{z}_{i}| < {z}_{1-\alpha /2k}$, then the null hypothesis can’t be rejected.

For checking the existence of outlying means of the individual series, the Mandel’s h statistic^49^ was used, implemented in ‘ILS’ package in R^50^.

Let *k* be the number of samples, ${\bar{x}}_{i},\,i\,=\,1,\,2,\,\mathrm{...},\,k,$ their means and $\bar{\bar{x}}$the overall mean. The Mandel’s h test statistics are:

22 $${h}_{i}=\frac{\overline{{x}_{i}}-\overline{\overline{x}}}{{[1/(k-1)\sum _{i=1}^{k}{(\overline{{x}_{i}}-\overline{\overline{x}})}^{2}]}^{1/2}}$$

and they have the same distribution for all $i\,=\,1,\,2,\,\mathrm{...},\,k.$

For example, for *i* = *k*, the critical values are given by:

23 $${h}_{k;1-\alpha /2}=\frac{(k-1){t}_{k-2;1-\alpha /2}}{{[k(k-2)+{t}_{k-2;1-\alpha /2}^{2}]}^{1/2}},$$

where ${t}_{k-2;1-\alpha /2}$ is the ($1-\alpha /2)\,-$quantile of the *t-*distribution with (*k* − 2) degrees of freedom^51^.

To test the hypothesis H_0_: *The variances of the individual series are identical*, against the alternative one *H*_*a*_*: At least one of the variances is not identical to the others*, the Brown-Forsythe test^52^, implemented in ‘lawstat’ package in R^53^ has been performed.

Let ${z}_{ij}\,=\,|{y}_{ij}-\overline{{y}_{j}}|,$where ${y}_{ij}$ is the element *i* in the group *j* and $\overline{{y}_{j}}$ is the median of group *j*. The Brown – Forsythe test statistic is

24 $$F=\frac{N-p}{p-1}\frac{\sum _{j=1}^{k}{n}_{j}{(\overline{{z}_{.j}}-\overline{{z}_{\mathrm{..}}})}^{2}}{\sum _{j=1}^{k}\sum _{i=1}^{{n}_{j}}{({z}_{ij}-{z}_{.j})}^{2}},$$

where *N* is the total number of observations, *k* is the number of groups, *n*_*j*_ is the number of observations in group *j*, $\overline{{z}_{.j}}$ is the mean of the elements *z*_*ij*_ in group *j*, and $\overline{{z}_{\mathrm{..}}}$ is the overall mean of the *z*_*ij*_.

The test rejects the hypothesis that the variances are equal at the significance level *α* if $F > {F}_{\alpha ,k-1,n-k}$, where ${F}_{\alpha ,k-1,n-k}$ is the upper critical value of the F distribution with (*k* – 1) and (*n* – *k*) degrees of freedom at the significance level of *α*. Alternatively, the null hypothesis is rejected if the p-value corresponding to the test is less than *α*.

### Availability statement

Data are available in the Supplementary Database1.

## Electronic supplementary material

Supplemetary Database 1

Supplementary Tables
