# Supplementary material for: Statistical analysis and estimation of the regional trend of aerosol size over the Arabian Gulf Region during 2002–2016
Source: Sci Rep. 2018 Jun 22;8:9571. doi: 10.1038/s41598-018-27727-0 (PMC6015092; doi:10.1038/s41598-018-27727-0)
Supplement: Supplementary file 2 — Supplementary Tables [file 41598_2018_27727_MOESM2_ESM.pdf]

Statistical analysis and estimation of the regional trend of aerosol size over the Arabian Gulf Region during 2002 -2016

Alina Barbulescu, Yousef Nazzal, Fares Howari

Supplementary Table S1. Modeling results

onth	Method I	Method II	Month	Method I	Method II	Month	Method I	Method II	Month	Method I	Method II	Month	Method I	Method II
Jul-02	51.49	52.71	May-05	48.52	49.71	Mar-08	52.19	51.82	Jan-11	53.00	53.44	Nov-13	63.23	59.46
Aug-02	50.29	50.95	Jun-05	48.56	47.05	Apr-08	42.78	43.37	Feb-11	54.45	51.98	Dec-13	68.84	68.18
Sep-02	62.45	61.77	Jul-05	46.32	45.78	May-08	39.02	39.25	Mar-11	41.50	41.71	Jan-14	67.98	68.98
Oct-02	65.87	66.72	Aug-05	52.80	53.34	Jun-08	41.36	43.17	Apr-11	38.84	38.63	Feb-14	61.86	64.00
Nov-02	69.87	69.49	Sep-05	59.51	59.95	Jul-08	42.11	41.31	May-11	44.38	45.62	Mar-14	49.32	51.79
Dec-02	57.34	57.41	Oct-05	70.61	71.71	Aug-08	43.77	44.26	Jun-11	46.14	45.66	Apr-14	45.16	44.93
Jan-03	68.81	70.12	Nov-05	65.38	64.54	Sep-08	52.26	50.75	Jul-11	51.27	50.19	May-14	49.05	48.81
Feb-03	58.24	55.81	Dec-05	69.62	70.44	Oct-08	62.99	62.97	Aug-11	62.91	62.67	Jun-14	46.54	48.03
Mar-03	51.97	52.78	Jan-06	55.47	54.42	Nov-08	68.63	68.46	Sep-11	61.23	61.52	Jul-14	52.81	50.62
Apr-03	54.94	61.77	Feb-06	58.37	59.77	Dec-08	68.84	68.63	Oct-11	67.27	66.62	Aug-14	61.20	62.42
May-03	39.67	37.55	Mar-06	57.86	57.80	Jan-09	62.67	61.97	Nov-11	61.12	64.06	Sep-14	64.66	65.24
Jun-03	49.76	46.94	Apr-06	63.93	59.06	Feb-09	53.17	50.73	Dec-11	57.98	56.62	Oct-14	64.93	65.57
Jul-03	48.41	50.01	May-06	42.39	43.95	Mar-09	52.76	50.85	Jan-12	53.15	51.53	Nov-14	66.31	66.97
Aug-03	50.89	49.72	Jun-06	52.29	54.37	Apr-09	47.07	45.05	Feb-12	49.93	48.00	Dec-14	72.34	73.90
Sep-03	58.29	58.04	Jul-06	43.36	43.15	May-09	42.47	44.43	Mar-12	41.65	41.35	Jan-15	56.47	55.46
Oct-03	64.75	63.46	Aug-06	46.18	45.79	Jun-09	44.51	44.89	Apr-12	37.78	40.53	Feb-15	58.86	59.31
Nov-03	57.08	56.73	Sep-06	60.99	60.29	Jul-09	38.83	39.32	May-12	43.49	43.21	Mar-15	40.80	40.31
Dec-03	50.57	47.91	Oct-06	69.31	68.48	Aug-09	41.21	42.67	Jun-12	46.42	46.48	Apr-15	45.34	44.68
Jan-04	60.09	59.26	Nov-06	68.06	68.90	Sep-09	58.25	58.01	Jul-12	46.23	45.48	May-15	45.82	42.88
Feb-04	58.91	58.48	Dec-06	63.25	64.72	Oct-09	62.97	63.59	Aug-12	57.40	57.56	Jun-15	41.87	41.88
Mar-04	62.33	62.52	Jan-07	63.46	60.96	Nov-09	64.43	64.72	Sep-12	71.01	71.55	Jul-15	56.03	52.55
Apr-04	49.78	48.33	Feb-07	58.11	56.30	Dec-09	68.48	68.71	Oct-12	71.77	73.81	Aug-15	56.35	57.67
May-04	43.49	39.58	Mar-07	49.75	48.49	Jan-10	55.95	56.56	Nov-12	65.10	63.20	Sep-15	63.90	65.45
Jun-04	47.93	48.45	Apr-07	40.74	45.15	Feb-10	54.07	54.94	Dec-12	60.12	60.31	Oct-15	66.12	66.99
Jul-04	50.33	49.25	May-07	40.62	38.96	Mar-10	48.67	44.33	Jan-13	64.73	63.06	Nov-15	65.71	65.66
Aug-04	53.38	52.84	Jun-07	41.66	44.21	Apr-10	44.52	43.25	Feb-13	55.21	56.29	Dec-15	59.83	59.48
Sep-04	57.08	54.43	Jul-07	45.00	44.25	May-10	41.11	40.33	Mar-13	43.92	45.07	Jan-16	70.98	66.33
Oct-04	68.39	67.84	Aug-07	54.78	55.15	Jun-10	42.48	43.80	Apr-13	37.40	38.46	Feb-16	57.75	55.56
Nov-04	63.23	63.25	Sep-07	59.07	58.56	Jul-10	51.63	52.01	May-13	42.35	44.35	Mar-16	60.68	61.59
Dec-04	56.04	54.92	Oct-07	73.48	71.77	Aug-10	59.27	58.65	Jun-13	46.03	44.96	Apr-16	43.48	44.28
Jan-05	58.59	56.69	Nov-07	66.99	69.16	Sep-10	65.48	63.78	Jul-13	48.90	47.51	May-16	46.18	49.03
Feb-05	59.28	58.32	Dec-07	63.80	63.00	Oct-10	73.83	73.74	Aug-13	57.05	57.10	Jun-16	44.15	44.40
Mar-05	59.00	60.67	Jan-08	60.64	63.00	Nov-10	65.73	66.54	Sep-13	65.45	65.08	Jul-16	46.77	54.50
Apr-05	49.70	49.83	Feb-08	53.73	53.92	Dec-10	62.09	64.37	Oct-13	72.65	72.65	Aug-16	64.78	64.61

Supplementary Table S2. Modeling errors

	MAD			MSE		
Point	Method I	Method II	Difference	Method I	Method II	Difference
Point 1	3.88	4.22	-0.34	5.37	5.79	-0.42
Point 2	3.56	3.84	-0.28	4.77	5.22	-0.45
Point 3	2.90	3.15	-0.24	3.50	3.82	-0.32
Point 4	2.78	3.13	-0.36	3.47	3.85	-0.39
Point 5	2.44	2.96	-0.52	3.14	3.69	-0.55
Point 6	2.23	2.66	-0.43	2.94	3.46	-0.52
Point 7	2.23	2.60	-0.37	2.91	3.41	-0.50
Point 8	2.17	2.62	-0.45	2.86	3.43	-0.58
Point 9	2.13	2.61	-0.48	2.82	3.44	-0.62
Point 10	2.15	2.66	-0.51	2.86	3.53	-0.66
Point 11	2.17	2.68	-0.51	2.90	3.55	-0.65
Point 12	2.03	2.54	-0.51	2.80	3.38	-0.58
Point 13	1.98	2.40	-0.42	2.67	3.16	-0.49
Point 14	1.97	2.31	-0.35	2.87	3.31	-0.45
Point 15	1.96	2.24	-0.28	2.63	2.98	-0.34
Point 16	2.07	2.19	-0.12	2.67	2.85	-0.18
Point 17	2.01	1.95	0.06	2.54	2.41	0.13
Point 18	2.00	1.94	0.06	2.73	2.51	0.22
Point 19	1.90	1.79	0.11	2.66	2.33	0.33
Point 20	1.93	1.63	0.30	2.66	2.20	0.46
Point 21	1.75	1.35	0.39	2.36	1.72	0.64
Point 22	1.81	1.32	0.49	2.51	1.76	0.76
Point 23	1.83	1.33	0.50	2.58	1.76	0.82
Point 24	1.95	1.40	0.55	2.64	1.79	0.84
Point 25	2.15	1.59	0.55	2.83	2.06	0.77
Point 26	2.47	2.06	0.41	3.13	2.62	0.51
Point 27	2.65	2.29	0.36	3.38	2.93	0.45
Point 28	3.07	2.70	0.37	3.87	3.51	0.36
Point 29	5.06	5.22	-0.16	6.88	7.17	-0.29
Point 30	3.89	4.17	-0.29	4.96	5.29	-0.33
Point 31	3.25	3.49	-0.24	3.99	4.45	-0.46
Point 32	2.77	3.26	-0.49	3.50	4.07	-0.57
Point 33	2.48	2.99	-0.52	3.28	3.93	-0.65
Point 34	2.25	2.75	-0.50	2.98	3.62	-0.64
Point 35	2.14	2.73	-0.58	2.85	3.50	-0.65
Point 36	2.09	2.60	-0.51	2.76	3.41	-0.65
Point 37	2.12	2.64	-0.52	2.83	3.45	-0.62
Point 38	2.07	2.59	-0.52	2.77	3.40	-0.63
Point 39	1.93	2.46	-0.53	2.64	3.26	-0.62
Point 40	1.95	2.41	-0.46	2.66	3.19	-0.52
Point 41	1.90	2.29	-0.39	2.47	2.94	-0.47
Point 42	1.92	2.20	-0.28	2.53	2.86	-0.33
Point 43	2.02	2.12	-0.11	2.59	2.80	-0.21

Point 44	1.95	1.95	0.00	2.41	2.40	0.01
Point 45	1.91	1.86	0.05	2.58	2.35	0.22
Point 46	1.84	1.70	0.14	2.43	2.12	0.31
Point 47	1.78	1.46	0.32	2.41	1.93	0.48
Point 48	1.78	1.29	0.49	2.43	1.70	0.74
Point 49	1.83	1.27	0.57	2.56	1.69	0.87
Point 50	1.89	1.21	0.68	2.66	1.67	0.98
Point 51	1.96	1.26	0.70	2.73	1.74	1.00
Point 52	2.04	1.39	0.65	2.68	1.85	0.84
Point 53	2.23	1.87	0.36	2.97	2.43	0.54
Point 54	2.54	2.26	0.28	3.44	2.95	0.49
Point 55	3.25	2.90	0.35	4.22	3.78	0.43
Point 56	5.71	5.98	-0.27	7.89	8.25	-0.36
Point 57	3.82	4.24	-0.42	4.73	5.27	-0.54
Point 58	3.08	3.58	-0.50	3.98	4.46	-0.48
Point 59	2.58	3.17	-0.59	3.56	4.12	-0.56
Point 60	2.22	2.66	-0.44	2.90	3.43	-0.52
Point 61	2.13	2.65	-0.53	2.87	3.46	-0.59
Point 62	2.16	2.68	-0.52	2.85	3.45	-0.60
Point 63	2.13	2.67	-0.54	2.92	3.53	-0.61
Point 64	2.06	2.69	-0.63	2.75	3.42	-0.67
Point 65	2.02	2.61	-0.59	2.68	3.32	-0.64
Point 66	1.95	2.48	-0.53	2.54	3.16	-0.62
Point 67	1.83	2.33	-0.49	2.41	2.94	-0.53
Point 68	1.91	2.20	-0.29	2.47	2.78	-0.31
Point 69	1.98	2.19	-0.21	2.54	2.73	-0.19
Point 70	1.89	1.95	-0.06	2.37	2.42	-0.05
Point 71	1.84	1.73	0.11	2.46	2.24	0.21
Point 72	1.75	1.69	0.06	2.41	2.17	0.24
Point 73	1.75	1.51	0.24	2.44	1.93	0.51
Point 74	1.74	1.33	0.42	2.49	1.77	0.72
Point 75	1.89	1.28	0.61	2.65	1.77	0.87
Point 76	2.02	1.34	0.67	2.82	1.82	1.00
Point 77	2.08	1.35	0.74	2.85	1.77	1.08
Point 78	2.11	1.30	0.81	2.86	1.73	1.13
Point 79	2.26	1.55	0.71	3.04	2.16	0.88
Point 80	2.58	2.08	0.50	3.45	2.88	0.57
Point 81	2.81	2.40	0.41	3.60	3.06	0.54
Point 82	3.51	3.98	-0.47	4.55	5.06	-0.51
Point 83	2.88	3.43	-0.55	3.84	4.38	-0.54
Point 84	2.27	2.72	-0.45	3.00	3.50	-0.50
Point 85	2.04	2.58	-0.53	2.80	3.42	-0.62
Point 86	2.02	2.52	-0.51	2.72	3.33	-0.61
Point 87	2.07	2.61	-0.55	2.73	3.41	-0.68
Point 88	2.02	2.50	-0.48	2.66	3.29	-0.64
Point 89	2.03	2.54	-0.51	2.66	3.29	-0.63
Point 90	1.92	2.54	-0.62	2.55	3.21	-0.66

Point 91	1.88	2.46	-0.58	2.49	3.11	-0.61
Point 92	1.86	2.31	-0.45	2.39	2.87	-0.48
Point 93	1.86	2.18	-0.33	2.42	2.75	-0.33
Point 94	1.87	1.94	-0.06	2.35	2.43	-0.07
Point 95	1.93	1.87	0.06	2.52	2.37	0.15
Point 96	1.99	1.83	0.16	2.60	2.31	0.28
Point 97	1.90	1.65	0.25	2.64	2.12	0.52
Point 98	1.85	1.47	0.39	2.62	1.91	0.71
Point 99	1.90	1.37	0.54	2.64	1.83	0.81
Point 100	2.01	1.24	0.77	2.79	1.77	1.02
Point 101	2.01	1.26	0.76	2.79	1.69	1.11
Point 102	2.09	1.27	0.82	2.84	1.69	1.15
Point 103	2.19	1.37	0.83	2.94	1.78	1.16
Point 104	2.32	1.67	0.65	3.03	2.21	0.83
Point 105	2.82	2.37	0.45	3.57	3.04	0.53
Point 106	4.77	4.43	0.34	6.86	6.38	0.48
Point 107	6.07	5.88	0.19	8.24	8.16	0.07
Point 108	3.44	3.44	0.00	4.34	4.57	-0.23
Point 109	3.00	3.02	-0.02	3.77	3.85	-0.08
Point 110	3.34	3.42	-0.07	4.32	4.55	-0.22
Point 111	4.53	4.63	-0.11	5.98	6.25	-0.27
Point 112	7.11	7.33	-0.22	9.63	10.08	-0.45
Point 113	3.41	3.84	-0.43	4.35	4.91	-0.56
Point 114	2.87	3.49	-0.62	3.94	4.60	-0.65
Point 115	2.49	3.02	-0.54	3.36	3.95	-0.58
Point 116	2.09	2.69	-0.59	2.93	3.54	-0.61
Point 117	2.09	2.63	-0.54	2.87	3.50	-0.62
Point 118	2.17	2.69	-0.52	2.90	3.62	-0.72
Point 119	2.03	2.56	-0.53	2.70	3.38	-0.68
Point 120	2.09	2.55	-0.46	2.67	3.26	-0.59
Point 121	2.06	2.59	-0.53	2.66	3.26	-0.61
Point 122	1.97	2.47	-0.50	2.55	3.15	-0.60
Point 123	1.92	2.43	-0.51	2.52	3.09	-0.57
Point 124	1.84	2.22	-0.39	2.39	2.78	-0.39
Point 125	1.84	2.05	-0.21	2.30	2.60	-0.29
Point 126	1.94	2.00	-0.06	2.42	2.51	-0.09
Point 127	1.97	1.88	0.09	2.55	2.37	0.18
Point 128	1.78	1.53	0.25	2.41	1.97	0.44
Point 129	1.72	1.43	0.29	2.47	1.84	0.64
Point 130	1.81	1.33	0.48	2.60	1.74	0.86
Point 131	1.91	1.28	0.63	2.71	1.79	0.92
Point 132	1.97	1.23	0.74	2.80	1.69	1.11
Point 133	2.02	1.21	0.80	2.76	1.62	1.14
Point 134	2.10	1.25	0.85	2.82	1.65	1.17
Point 135	2.32	1.72	0.60	3.17	2.29	0.88
Point 136	3.00	2.63	0.37	3.91	3.41	0.50
Point 137	4.26	4.01	0.25	5.61	5.28	0.33

Point 138	3.65	3.77	-0.12	4.75	4.96	-0.21
Point 139	3.55	3.66	-0.10	4.52	4.68	-0.16
Point 140	4.11	4.20	-0.09	5.16	5.34	-0.18
Point 141	3.45	3.68	-0.23	4.25	4.58	-0.33
Point 142	2.80	3.32	-0.51	3.59	4.16	-0.57
Point 143	2.77	3.31	-0.54	3.60	4.22	-0.62
Point 144	2.89	3.41	-0.52	4.45	5.25	-0.80
Point 145	3.57	4.00	-0.43	5.35	6.02	-0.66
Point 146	3.93	4.37	-0.44	5.66	6.30	-0.63
Point 147	3.35	3.73	-0.38	4.89	5.54	-0.65
Point 148	2.51	2.99	-0.48	3.43	4.14	-0.71
Point 149	2.44	2.86	-0.42	3.34	3.99	-0.65
Point 150	2.27	2.63	-0.36	2.82	3.40	-0.58
Point 151	2.02	2.55	-0.52	2.55	3.16	-0.61
Point 152	1.75	2.23	-0.47	2.31	2.91	-0.59
Point 153	1.58	1.89	-0.30	2.04	2.40	-0.36
Point 154	1.70	1.95	-0.25	2.15	2.41	-0.26
Point 155	1.92	1.91	0.01	2.46	2.38	0.08
Point 156	1.87	1.80	0.07	2.54	2.30	0.24
Point 157	1.77	1.63	0.14	2.54	2.14	0.40
Point 158	1.83	1.47	0.35	2.62	1.91	0.71
Point 159	1.71	1.29	0.43	2.45	1.69	0.76
Point 160	1.73	1.14	0.60	2.50	1.51	0.98
Point 161	1.90	1.17	0.73	2.62	1.57	1.05
Point 162	2.01	1.19	0.83	2.74	1.66	1.08
Point 163	2.10	1.43	0.67	2.83	1.91	0.92
Point 164	2.12	1.64	0.48	2.88	2.17	0.71
Point 165	2.29	1.88	0.41	2.94	2.40	0.54
Point 166	2.33	2.07	0.26	3.13	2.66	0.47
Point 167	2.77	2.42	0.34	3.49	3.09	0.41
Point 168	2.66	2.39	0.27	3.42	3.12	0.30
Point 169	2.51	2.35	0.16	3.34	3.13	0.21
Point 170	3.54	3.43	0.12	4.67	4.51	0.16
Point 171	4.20	4.17	0.03	5.43	5.43	-0.01
Point 172	4.51	4.67	-0.16	5.77	5.85	-0.08
Point 173	4.42	4.51	-0.09	5.58	5.66	-0.08
Point 174	3.82	3.85	-0.04	5.00	5.07	-0.07
Point 175	4.00	4.08	-0.08	5.34	5.39	-0.06
Point 176	4.94	5.00	-0.06	6.49	6.67	-0.18
Point 177	3.76	4.00	-0.24	4.71	5.14	-0.44
Point 178	2.87	3.37	-0.50	3.84	4.35	-0.51
Point 179	2.78	3.32	-0.54	3.82	4.34	-0.52
Point 180	3.47	3.84	-0.38	4.56	4.99	-0.43
Point 181	4.51	4.72	-0.20	6.14	6.33	-0.19
Point 182	3.39	3.69	-0.30	4.29	4.72	-0.42
Point 183	2.22	2.70	-0.48	2.82	3.45	-0.63
Point 184	2.17	2.62	-0.45	2.89	3.49	-0.60

Point 185	2.02	2.48	-0.46	2.68	3.27	-0.59
Point 186	1.85	2.35	-0.50	2.46	3.06	-0.61
Point 187	1.65	2.03	-0.38	2.18	2.64	-0.46
Point 188	1.68	1.93	-0.25	2.24	2.47	-0.24
Point 189	1.88	1.91	-0.02	2.41	2.39	0.02
Point 190	1.90	1.92	-0.02	2.54	2.38	0.15
Point 191	1.78	1.67	0.11	2.40	2.12	0.28
Point 192	1.84	1.60	0.24	2.52	2.04	0.48
Point 193	1.79	1.40	0.40	2.52	1.81	0.71
Point 194	1.75	1.25	0.50	2.49	1.69	0.80
Point 195	1.85	1.21	0.64	2.60	1.71	0.89
Point 196	1.91	1.26	0.65	2.65	1.76	0.89
Point 197	1.89	1.31	0.57	2.57	1.75	0.82
Point 198	1.89	1.35	0.54	2.50	1.71	0.79
Point 199	1.99	1.52	0.47	2.59	1.93	0.66
Point 200	1.88	1.50	0.39	2.50	1.89	0.61
Point 201	1.89	1.52	0.37	2.45	1.89	0.57
Point 202	2.13	1.78	0.35	2.71	2.26	0.45
Point 203	2.31	1.94	0.37	2.88	2.43	0.45
Point 204	2.47	2.28	0.19	3.19	2.76	0.42
Point 205	2.68	2.56	0.12	3.36	3.18	0.18
Point 206	2.84	2.79	0.05	3.56	3.45	0.11
Point 207	3.07	3.14	-0.07	3.88	3.88	0.00
Point 208	3.67	3.66	0.02	4.73	4.69	0.04
Point 209	4.52	4.59	-0.07	5.90	6.03	-0.12
Point 210	3.41	3.77	-0.35	4.25	4.83	-0.58
Point 211	2.67	3.02	-0.35	3.36	3.82	-0.46
Point 212	2.50	2.96	-0.46	3.25	3.77	-0.51
Point 213	2.49	2.85	-0.36	3.21	3.76	-0.55
Point 214	2.59	3.02	-0.44	3.47	3.99	-0.52
Point 215	2.63	3.00	-0.36	3.63	4.10	-0.47
Point 216	2.68	2.98	-0.30	3.44	3.94	-0.50
Point 217	2.25	2.76	-0.51	2.95	3.58	-0.63
Point 218	2.11	2.57	-0.46	2.76	3.39	-0.64
Point 219	2.05	2.56	-0.52	2.69	3.39	-0.70
Point 220	2.04	2.56	-0.52	2.71	3.40	-0.69
Point 221	1.88	2.45	-0.58	2.53	3.21	-0.67
Point 222	1.72	2.23	-0.51	2.30	2.86	-0.56
Point 223	1.69	2.11	-0.43	2.20	2.65	-0.45
Point 224	1.89	2.15	-0.25	2.49	2.69	-0.20
Point 225	1.85	1.94	-0.09	2.38	2.44	-0.05
Point 226	1.77	1.75	0.02	2.33	2.25	0.07
Point 227	1.76	1.58	0.18	2.34	2.06	0.28
Point 228	1.79	1.43	0.36	2.47	1.85	0.62
Point 229	1.93	1.36	0.57	2.62	1.86	0.76
Point 230	1.85	1.32	0.53	2.57	1.73	0.84
Point 231	1.74	1.22	0.52	2.37	1.57	0.80

Point 232	1.74	1.25	0.49	2.34	1.59	0.75
Point 233	1.87	1.29	0.57	2.43	1.65	0.78
Point 234	2.03	1.55	0.48	2.67	1.98	0.70
Point 235	2.02	1.57	0.45	2.64	1.97	0.67
Point 236	2.05	1.59	0.45	2.67	2.01	0.66
Point 237	1.99	1.71	0.29	2.60	2.15	0.45
Point 238	2.05	1.78	0.27	2.64	2.21	0.43
Point 239	2.32	2.17	0.14	3.00	2.71	0.29
Point 240	2.68	2.59	0.09	3.41	3.25	0.16
Point 241	2.91	2.81	0.10	3.61	3.51	0.10
Point 242	3.21	3.20	0.02	4.06	4.00	0.06
Point 243	4.21	4.14	0.08	5.50	5.42	0.08
Point 244	5.05	5.23	-0.18	6.71	6.93	-0.22
Point 245	4.00	4.34	-0.34	5.40	6.00	-0.59
Point 246	2.81	3.15	-0.34	3.52	4.01	-0.49
Point 247	2.42	2.88	-0.45	3.16	3.69	-0.53
Point 248	2.40	2.87	-0.47	3.10	3.66	-0.56
Point 249	2.24	2.73	-0.48	2.94	3.57	-0.63
Point 250	2.14	2.59	-0.45	2.84	3.42	-0.59
Point 251	1.99	2.60	-0.60	2.63	3.31	-0.67
Point 252	2.02	2.64	-0.61	2.64	3.40	-0.76
Point 253	1.99	2.61	-0.62	2.60	3.40	-0.80
Point 254	2.12	2.69	-0.56	2.71	3.46	-0.75
Point 255	2.05	2.65	-0.60	2.70	3.45	-0.75
Point 256	1.85	2.46	-0.61	2.53	3.20	-0.68
Point 257	1.74	2.25	-0.51	2.33	2.93	-0.60
Point 258	1.72	2.10	-0.38	2.17	2.67	-0.49
Point 259	1.86	2.14	-0.28	2.41	2.65	-0.24
Point 260	1.89	1.94	-0.06	2.49	2.50	-0.01
Point 261	1.82	1.85	-0.03	2.37	2.37	0.01
Point 262	1.83	1.75	0.08	2.41	2.20	0.20
Point 263	1.80	1.56	0.24	2.42	1.99	0.43
Point 264	1.84	1.44	0.39	2.47	1.87	0.60
Point 265	1.88	1.40	0.48	2.45	1.75	0.70
Point 266	1.90	1.42	0.48	2.47	1.77	0.70
Point 267	1.99	1.47	0.52	2.62	1.88	0.74
Point 268	1.92	1.33	0.59	2.51	1.68	0.83
Point 269	1.90	1.43	0.47	2.49	1.79	0.69
Point 270	1.87	1.46	0.42	2.50	1.86	0.64
Point 271	1.84	1.52	0.32	2.44	1.96	0.48
Point 272	2.03	1.79	0.24	2.65	2.28	0.37
Point 273	2.14	1.98	0.16	2.86	2.52	0.34
Point 274	2.36	2.23	0.13	3.08	2.81	0.27
Point 275	2.78	2.66	0.13	3.59	3.52	0.07
Point 276	3.26	3.33	-0.07	4.17	4.29	-0.12
Point 277	4.51	4.66	-0.16	5.91	6.10	-0.19
Point 278	5.92	6.09	-0.17	7.81	8.00	-0.18

Point 279	4.22	4.62	-0.40	5.71	6.25	-0.54
Point 280	3.01	3.33	-0.31	3.80	4.30	-0.50
Point 281	2.46	2.85	-0.39	3.10	3.63	-0.54
Point 282	2.40	2.85	-0.45	3.02	3.60	-0.58
Point 283	2.35	2.87	-0.52	3.04	3.66	-0.62
Point 284	2.19	2.69	-0.50	2.87	3.48	-0.61
Point 285	2.17	2.70	-0.53	2.86	3.48	-0.62
Point 286	2.28	2.78	-0.50	2.89	3.56	-0.67
Point 287	2.30	2.78	-0.48	2.91	3.65	-0.74
Point 288	2.10	2.64	-0.54	2.76	3.50	-0.75
Point 289	2.12	2.62	-0.50	2.78	3.51	-0.73
Point 290	2.00	2.53	-0.53	2.65	3.31	-0.66
Point 291	1.86	2.35	-0.50	2.50	3.06	-0.56
Point 292	1.90	2.34	-0.44	2.53	3.01	-0.48
Point 293	1.87	2.11	-0.24	2.40	2.73	-0.33
Point 294	1.90	1.99	-0.09	2.47	2.56	-0.09
Point 295	1.83	1.93	-0.10	2.45	2.53	-0.08
Point 296	1.83	1.82	0.01	2.39	2.30	0.08
Point 297	1.83	1.73	0.11	2.49	2.17	0.33
Point 298	2.03	1.70	0.33	2.61	2.18	0.43
Point 299	2.25	1.86	0.38	2.89	2.30	0.59
Point 300	2.81	2.35	0.46	3.94	3.32	0.62
Point 301	3.45	3.12	0.32	4.61	4.10	0.51
Point 302	3.76	3.48	0.28	5.07	4.60	0.47
Point 303	3.20	2.86	0.33	4.12	3.68	0.44
Point 304	2.65	2.31	0.34	3.37	2.96	0.41
Point 305	2.48	2.19	0.30	3.13	2.74	0.39
Point 306	2.35	2.20	0.15	3.02	2.76	0.27
Point 307	2.48	2.29	0.18	3.15	2.96	0.19
Point 308	2.84	2.80	0.04	3.66	3.48	0.18
Point 309	3.34	3.27	0.07	4.25	4.15	0.10
Point 310	4.16	4.14	0.02	5.66	5.54	0.12
Point 311	4.61	4.93	-0.32	6.18	6.55	-0.37
Point 312	3.24	3.53	-0.28	4.08	4.44	-0.36
Point 313	2.55	2.98	-0.44	3.36	3.88	-0.52
Point 314	2.49	2.95	-0.46	3.35	3.85	-0.50
Point 315	2.29	2.82	-0.54	3.05	3.63	-0.58
Point 316	2.31	2.87	-0.55	3.04	3.67	-0.64
Point 317	2.30	2.86	-0.56	3.03	3.72	-0.68
Point 318	2.30	2.90	-0.61	3.03	3.77	-0.74
Point 319	2.87	3.38	-0.51	4.20	4.99	-0.79
Point 320	3.43	3.78	-0.35	4.80	5.44	-0.64
Point 321	3.54	3.95	-0.41	4.81	5.49	-0.68
Point 322	2.79	3.13	-0.34	3.83	4.42	-0.59
Point 323	2.51	2.87	-0.36	3.58	4.21	-0.63
Point 324	2.21	2.59	-0.38	3.15	3.71	-0.56
Point 325	1.95	2.29	-0.34	2.58	2.89	-0.31

Point 326	1.88	2.08	-0.20	2.42	2.67	-0.24
Point 327	1.98	2.14	-0.15	2.70	2.78	-0.07
Point 328	1.96	1.93	0.03	2.62	2.56	0.05
Point 329	2.03	1.99	0.03	2.82	2.60	0.21
Point 330	2.47	2.23	0.25	3.25	2.85	0.39
Point 331	3.20	2.96	0.24	4.13	3.78	0.35
Point 332	3.60	3.41	0.19	4.67	4.52	0.15
Point 333	2.64	2.57	0.07	3.63	3.43	0.20
Point 334	2.89	2.88	0.01	3.72	3.53	0.19
Point 335	3.41	3.43	-0.03	4.44	4.32	0.13
Point 336	4.23	4.20	0.03	5.56	5.51	0.05
Point 337	5.91	5.93	-0.01	8.07	8.11	-0.04
Point 338	3.76	3.92	-0.17	5.13	5.21	-0.08
Point 339	2.85	3.19	-0.33	3.71	4.11	-0.40
Point 340	2.79	3.15	-0.35	3.69	4.13	-0.44
Point 341	2.64	3.06	-0.42	3.51	4.02	-0.51
Point 342	2.93	3.39	-0.46	4.25	4.81	-0.57
Point 343	2.84	3.27	-0.43	3.88	4.44	-0.56
Point 344	2.79	3.14	-0.36	3.90	4.42	-0.52
Point 345	3.67	3.97	-0.30	4.61	5.06	-0.45
Point 346	5.32	5.86	-0.54	7.17	7.78	-0.61
Point 347	3.46	3.90	-0.44	4.40	4.87	-0.47
Point 348	2.74	3.12	-0.38	3.53	4.03	-0.50
Point 349	2.15	2.47	-0.32	2.80	3.14	-0.35
Point 350	2.01	2.22	-0.21	2.61	2.88	-0.27
Point 351	2.06	2.22	-0.16	2.74	2.83	-0.09
Point 352	2.04	2.14	-0.10	2.75	2.79	-0.04
Point 353	2.11	2.06	0.06	2.83	2.76	0.07
Point 354	2.37	2.30	0.07	3.20	2.95	0.25
Point 355	2.84	2.69	0.15	3.74	3.52	0.23
Point 356	3.46	3.36	0.10	4.64	4.42	0.22
Point 357	5.03	4.95	0.07	6.65	6.48	0.17
Point 358	3.35	3.20	0.15	4.30	4.13	0.18
Point 359	2.75	2.73	0.02	3.63	3.51	0.12
Point 360	2.99	3.05	-0.06	3.95	3.95	0.00
Point 361	4.21	4.31	-0.10	5.50	5.51	-0.01
Point 362	4.52	4.75	-0.23	5.92	6.14	-0.22
Point 363	2.86	3.07	-0.22	3.58	3.95	-0.37
Point 364	2.79	3.13	-0.35	3.66	4.15	-0.49
Point 365	3.18	3.66	-0.48	4.26	4.72	-0.47
Point 366	3.31	3.79	-0.48	4.38	4.92	-0.54
Point 367	3.51	3.90	-0.39	4.79	5.28	-0.49
Point 368	3.26	3.58	-0.32	4.26	4.71	-0.46
Point 369	3.54	3.89	-0.35	4.55	5.04	-0.49
Point 370	4.32	4.64	-0.32	5.55	5.85	-0.29
Point 371	3.14	3.40	-0.26	4.23	4.53	-0.29
Point 372	2.75	3.00	-0.25	3.69	4.00	-0.32

Point 373	2.43	2.77	-0.35	3.12	3.50	-0.38
Point 374	2.25	2.51	-0.25	2.93	3.15	-0.23
Point 375	2.11	2.17	-0.07	2.63	2.68	-0.05
Point 376	2.08	2.15	-0.07	2.68	2.73	-0.05
Point 377	2.00	2.08	-0.08	2.54	2.57	-0.03
Point 378	1.96	1.93	0.03	2.56	2.43	0.12
Point 379	1.97	1.93	0.04	2.56	2.42	0.14
Point 380	2.15	2.02	0.13	2.85	2.57	0.28
Point 381	2.31	2.14	0.17	2.97	2.64	0.33
Point 382	2.51	2.40	0.11	3.29	3.00	0.29
Point 383	2.41	2.32	0.09	3.12	2.82	0.31
Point 384	2.62	2.40	0.22	3.35	2.98	0.37
Point 385	2.87	2.57	0.30	3.58	3.27	0.32
Point 386	3.14	2.89	0.25	4.02	3.65	0.37
Point 387	4.07	4.08	-0.01	5.25	5.10	0.15
Method I more performant than Method II			**223**			**218**
